# USING MULTI-LEVEL MODELING TO EXAMINE RACIAL DISPARITIES IN PALLIATIVE CARE AMONG OLDER ADULTS WITH HEART FAILURE

**DOI:** 10.1093/geroni/igad104.3689

**Published:** 2023-12-21

**Authors:** Ling Han, Erica Abel, Yan Zhan, Kathleen Akgün, Shelli Feder

**Affiliations:** Yale School of Medicine, New Haven, Connecticut, United States; VA Connecticut Healthcare System, West Haven, Connecticut, United States; Yale School of Nursing, New Haven, Connecticut, United States; VA Connecticut Healthcare System, West Haven, Connecticut, United States; Yale School of Nursing, New Haven, Connecticut, United States

## Abstract

Studies have found persistent disparities in guideline-concordant cares for heart failure among patients who self-identify as Black. We investigated how the guideline-concordant specialist palliative care (SPC) varied with race among older adults with advanced heart failure (AHF). We identified 16,464 older adults aged ≥65y (median: 74y) with AHF by ICD-9/10 codes seen between 1/1/2018-7/1/2020 in 83 Veterans Affairs Medical Centers (VAMC). In conventional logistic regression without accounting for between-VAMC variation, Black (28%) race was less likely to receive SPC services than White (70%) race [adjusted odds ratio (aOR) of 0.91 (95% Confidence Intervals (CI): 0.84,0.99); predicted probability: 27.6% vs. 29.5%], independent of age, sex and comorbidity. This association diminished to null [aOR: 0.99 (95% CI: 0.90,1.08)] after adding a random intercept for VAMCs (multi-level model (MLM)). Expanding the MLM with specific VAMC characteristics (SPC team staffing (p< 0.05), facility complexity and urbanicity) as fixed effects improved overall model fit (area under ROC: 0.68 vs 0.61, p< 0.05), and accounted for 17% of the VAMC-level variance (intraclass correlation coefficient: 0.069 vs 0.083). There were no cross-level interactions between race and VAMC characteristics. These findings suggest that variation in the individual probability of receiving SPC between Black and White race in this older AHF cohort may be partially explained by between-VAMC differences in SPC staffing and other facility characteristics. To promote equitable access to SPC for older adults with AHF, further research is needed to examine service delivery characteristics and broader contextual determinants of health, such as area socioeconomic disparity of healthcare facilities

